# First-in-Human Biodistribution and Dosimetry of [^11^C]Trimethoprim

**DOI:** 10.1007/s11307-025-02064-7

**Published:** 2025-11-22

**Authors:** Anthony J. Young, Robert K. Doot, Joshua K. Cho, Jonathan M. Pham, Alvaro A. Ordonez, Andres F. del Castillo, Tiffany L. Dominguez, Supritha Dugyala, Erin K. Schubert, Hsiaoju Lee, Austin R. Pantel, Robert H. Mach, David A. Mankoff, Mark A. Sellmyer

**Affiliations:** 1 Department of Radiology, Perelman School of Medicine, University of Pennsylvania, 813 A Stellar-Chance Labs, 422 Curie Boulevard, Philadelphia, PA 19104-6059 USA; 2 Department of Biochemistry and Biophysics, Perelman School of Medicine, University of Pennsylvania, Philadelphia, PA USA

**Keywords:** Trimethoprim, Reporter gene, Infection, PET/CT, Biodistribution, Dosimetry

## Abstract

**Abstract:**

Trimethoprim (TMP) is a reversible inhibitor of the prokaryotic enzyme dihydrofolate reductase (DHFR) used for the treatment or prophylaxis of bacterial infections. [^11^C]trimethoprim ([^11^C]TMP) is a positron emission tomography (PET) imaging isotopologue of TMP. TMP binds with 30,000-fold greater affinity to bacterial DHFR over the homologous mammalian enzyme *in vitro*, suggesting [^11^C]TMP may selectively accumulate in tissues with cells expressing bacterial DHFR. This study characterizes the biodistribution and dosimetry of [^11^C]TMP, informing its use in imaging bacterial infections and tracking mammalian cells expressing eDHFR as a reporter gene.

**Methods:**

Four males with suspected infection, aged 59 ± 10 years old (mean ± SD) received 3 serial PET/CT scans after injection of 346 ± 305 MBq (range 129–797 MBq) of [^11^C]TMP. Organ activities were measured in MIM v6.7, including brain, kidneys, spleen, liver, heart, lungs, bladder, intestines, gallbladder, pancreas, thyroid, and red marrow. Dosimetry calculations were performed in Olinda | EXM v1.1. Additionally, a dynamic whole-body PET/CT scan was performed on a separate participant. The associated trial was registered as NCT03424525.

**Results:**

[^11^C]TMP injections were well tolerated with no adverse events. The average injected activity of 346 MBq of [^11^C]TMP yielded an estimated average dose of 4.9 mSv in the highest uptake organ (liver), 4.1 mSv in the spleen, and an effective dose of 1.6 mSv. Suspected sites of infection displayed uptake above background.

**Conclusion:**

[^11^C]TMP PET was safe and demonstrated low background uptake in most tissues. The data suggests feasibility for evaluation of varied bacterial infections, including musculoskeletal infections. Absorbed doses allow multiple [^11^C]TMP PET scans each year within Radioactive Drug Research Committee (RDRC) limits, potentially enabling monitoring of infections and treatment response.

**Supplementary Information:**

The online version contains supplementary material available at 10.1007/s11307-025-02064-7.

## Introduction

Definitive diagnosis of bacterial infection has traditionally been achieved through tissue biopsy or fluid sampling, with subsequent culture necessary to verify diagnoses and identify bacterial strains. These invasive sampling methods are limited to accessible sites and risk transferring bacteria to surrounding tissues, missing active infection, and misidentifying unculturable bacteria [1, 2]. Non-invasive imaging, including structural modalities like computed tomography (CT), magnetic resonance imaging (MRI), and ultrasound imaging, as well as functional imaging using positron emission tomography (PET) or single-photon emission computed tomography (SPECT), have also been used to diagnose and monitor infection throughout the body [3]. However, imaging tools currently have limited specificity in diagnosing bacterial infection; for example, gallium-67 citrate SPECT, radiolabeled leukocyte (WBC) scans, and [^18^F]fluorodeoxyglucose (FDG) PET, all image the immune reaction to infection rather than the bacterial infection. Thus, these techniques are prone to false positives from other infections (e.g. viral), sterile inflammation, and malignancy.

Given the limitations in current imaging approaches, bacteria-specific metabolic imaging agents have been developed, including [^18^F]fluorodeoxysorbitol (FDS), [^11^C]para-aminobenzoic acid (PABA), D-amino acids, and ligands for the maltose transporter in bacteria [4–9]. Additional efforts have evaluated radiolabeled agents targeting the bacterial siderophore-mediated iron transport system [10, 11]. We have focused on the bacterial dihydrofolate reductase (DHFR) enzyme as a target for bacteria-specific imaging, as the protein is structurally distinct from mammalian DHFR. Trimethoprim (TMP) is a high affinity (IC50 = 5 nM) inhibitor of bacterial DHFR widely used in the treatment of bacterial infections [12, 13]. TMP has been reported to reversibly bind to the *Escherichia coli* homolog*, e*DHFR, with a dissociation rate constant ranging from 0.28 to 3.7 s^−1^ [14]. TMP radiotracers are radiolabeled PET imaging probes derived from TMP that have been studied for use in multiple applications including imaging genetically engineered cells, such as chimeric antigen receptor (CAR) T cells [15, 16], in addition to selective imaging of bacterial infection [13, 17].

Prior *in vitro* studies have confirmed that most bacteria, even in the setting of therapeutic TMP resistance, will retain TMP radiotracers [13, 17]. Findings demonstrating [^11^C]TMP utility for imaging bacterial infection have been reported previously [17]. However, a detailed understanding of the tracer’s biodistribution in multiple tissues is necessary to support its clinical translation. Here, we describe findings from the biodistribution and dosimetry cohort. We hypothesize [^11^C]TMP will have a favorable biodistribution for regular monitoring of disease state and progression.

## Materials and Methods

### Imaging Study Design

Subjects with localized known or suspected infection were recruited and provided informed consent according to guidelines of the University of Pennsylvania Institutional Review Board. Subjects undergoing TMP treatment within 48 h of baseline imaging were excluded from the study. The full study protocols are available at clinicaltrials.gov, NCT03424525 and were performed under the authority of the University of Pennsylvania Radioactive Drug Research Committee. Demographics are provided in Table 1.

**Table 1:** Subject characteristics at baseline [^11^C]TMP PET scan

ID	Sex	Age (y)	Height (cm)	Weight (kg)	IA* (MBq)	Disease	Antibiotics at time of baseline PET imaging	Bacteria isolated from infection site
7	M	65	183	120	217	Knee joint infection	None	*Staphylococcus aureus* (MRSA)^‡^
8	M	69	183	103	239	Knee joint infection	Amoxicillin	N/A
11	M	47	187	107	797	Charcot arthropathy	None	N/A
12^†^	M	54	168	84	129	Osteomyelitis/septic arthritis	Vancomycin	*Staphylococcus aureus* (MSSA)^§^

^*^Injected [^11^C]TMP activity

^†^Images previously published [8]

^‡^Methicillin resistant *Staphylococcus aureus* (MRSA), identified from tissue

^§^Methicillin susceptible *Staphylococcus aureus* (MSSA), identified from fluid aspirate

### Imaging Protocol

**Fig. 1:**
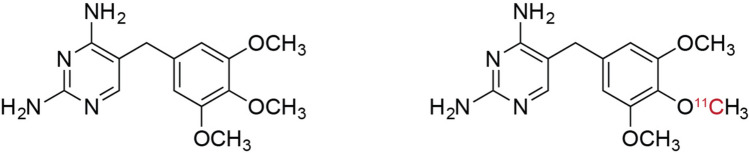
Structures of trimethoprim (TMP, left) and [^11^C]TMP (right)

**Fig. 2:**
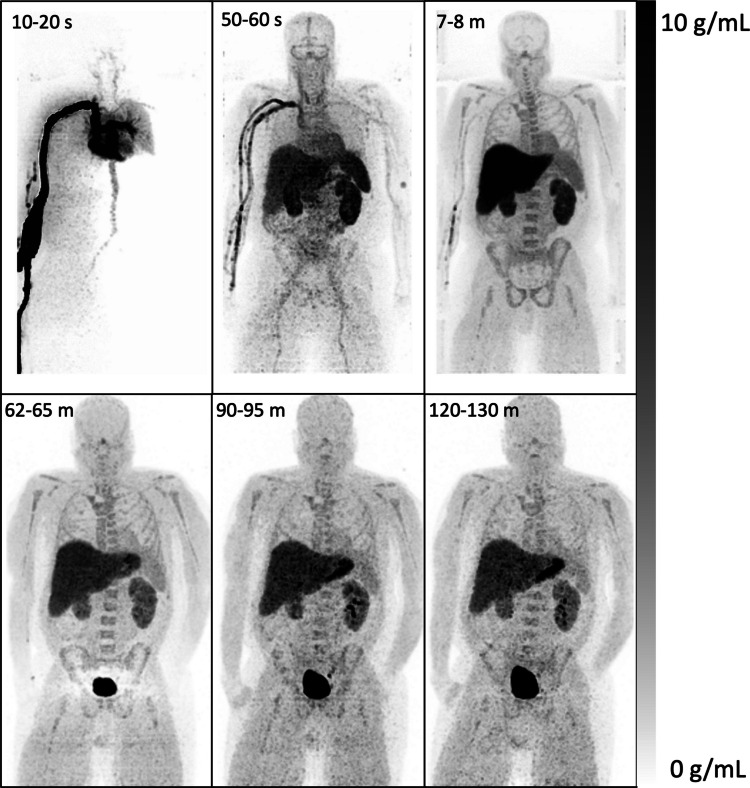
Dynamic PET maximum-intensity pixel images of [^11^C]TMP in a 52 year old male without active infection. Images were acquired on a whole-body PET/CT scanner following injection of 503 MBq of [^11^C]TMP. Image acquisition times post-injection are listed in the upper left corner

The structures of TMP and [^11^C]TMP are shown in Fig. 1. The radiotracer was synthesized as described previously [15], with a radiochemical purity of 97.2 ± 1.8% and molar activity of 43.1 ± 29.6 GBq/µmol (1165 ± 799 mCi/µmol), range 3.0–65.5 GBq/µmol (82–1772 mCi/µmol). Three serial whole-body PET (head to mid-thigh) and up to 3 low dose CT attenuation scans in a Philips Ingenuity PET/CT scanner (Philips Healthcare, Andover, MA) [18] were performed following injection of [^11^C]TMP (346 ± 305 MBq, 129–797 MBq (9.3 mCi, 3.5–21.5 mCi)). PET scans were acquired from head to mid-thigh over 15 min in 10 overlapping bed positions at 90 s per bed position with ~ 50% bed overlap. The first PET scan was started immediately following injection, followed by a second scan at 20 ± 3 min post-injection, and a third scan at 63 ± 4 min post-injection. Suspected infections outside of the dosimetry scan field of view were optionally imaged following the acquisition of the final dosimetry scan. Subjects had the option to void bladders between the second and third PET/CT scans and following the last scan, without urine collection. Images were reconstructed using a list-mode time-of-flight ordered subsets expectation maximization algorithm (TOF-OSEM) [18]. An additional participant with fever of unknown origin was injected with 503 MBq of [^11^C]TMP and scanned on a whole-body PET/CT scanner (PennPET Explorer) [19, 20]. These images were reconstructed using TOF-OSEM, but were not included in the quantitative dosimetry results (Fig. 2).

### Image Analysis

Total activity in the brain, gallbladder, heart, intestines, kidneys, liver, lungs, pancreas, spleen, thyroids, and urinary bladder was assessed via PET with MIM v6.7 (MIM Software Inc.), using co-registered CT images to delineate regions of interest. To enable estimation of total activity in red bone marrow, tracer uptake and volume were measured based on activity concentrations in the 2nd to 4th lumbar vertebrae of each subject. Using a red marrow density [21] of 1.03 g/mL and assuming that red marrow weight is 1.6% of total body weight [22], total red marrow activity was calculated for each subject. Time activity curves for organs were generated and in most cases fit to mono- or bi-exponential functions using OLINDA | EXM v1.1 software RRID:SCR_026835 [23]. For time activity curves that were poorly fit by exponential functions, a Riemann Sum was used to calculate numbers of disintegrations occurring in the organs. Dosimetry estimates were calculated using the Standard Adult Male phantom option in OLINDA.

## Results

### Subject Characteristics and Scanning

Characteristics of the dosimetry participants are shown in Table 1. Four male subjects were imaged as part of this study, which was primarily focused on evaluation of biodistribution and dosimetry. Three of the enrolled subjects had suspected localized infections. In two of them, the infection involved the prosthetic knee joint (subjects 7 and 8), and cultures of the knee joint fluid were positive for methicillin-resistant *Staphylococcus aureus* in one of them (subject 7). An additional participant (subject 12) had culture positive osteomyelitis as previously described [17]. Subject 11 was diagnosed with Charcot arthropathy with negative cultures of the affected ankle joint. An additional subject with fever of unknown origin and negative blood cultures was dynamically scanned under a separate imaging protocol using the total-body PennPET Explorer scanner, which allowed for late timepoint imaging (e.g., 120 min) with [^11^C]TMP given the sensitivity of the long-axis scanner (Fig. 2). Subjects in the dosimetry cohort scanned on the Philips Ingenuity PET/CT scanner had good image quality for both high and moderate-low injected activities (Suppl. Fig. 1 and Suppl. Fig. 2**,** respectively). [^11^C]TMP scans were well tolerated, with no adverse events observed in scanning or required follow-up.

### Lesion Uptake

**Fig. 3:**
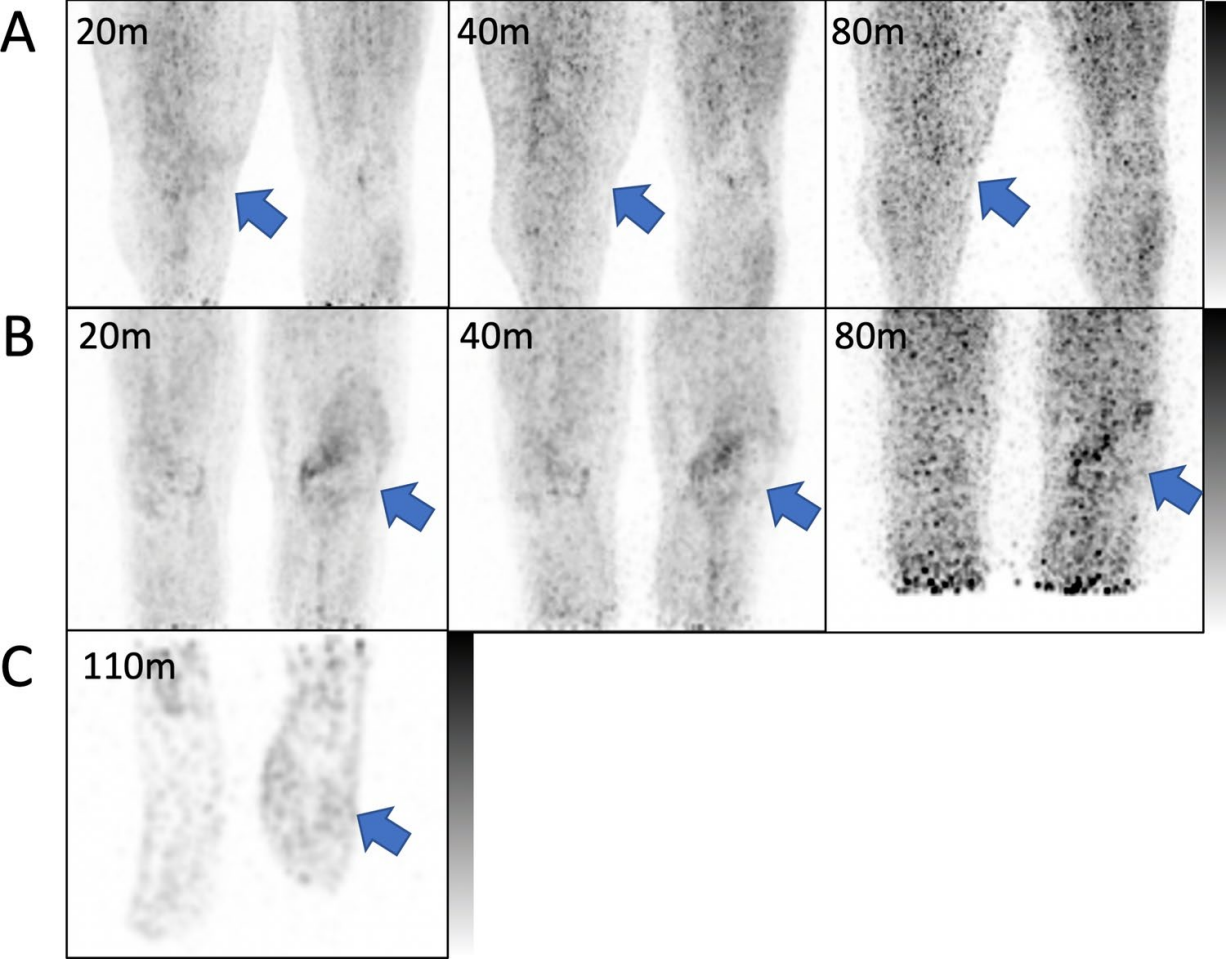
Maximum intensity projections of [^11^C]TMP scans of suspected or confirmed infections. **A** Subject 7 with bilateral knee implants and suspected right knee joint infection at 20, 40, and 80 min post-injection. **B** Subject 8 with bilateral knee implants and suspected left knee joint infection at 20, 40, and 80 min post-injection. **C** Subject 11 with left foot Charcot arthropathy at 110 min post-injection, following dosimetry scans. Images are scaled from 0–5 g/mL

Suspected or confirmed infectious lesions had mildly increased [^11^C]TMP uptake compared to the contralateral limb and/or surrounding normal tissue with a maximum standardized uptake value (SUV_max_) ratio of affected versus unaffected tissue of 1.3 ± 0.21 at 80–110 min post-injection (Fig. 3 and Suppl. Fig. 3). In the two subjects with prosthetic knee joints, [^11^C]TMP PET signal was mildly higher surrounding the affected prosthesis compared to the contralateral site (Fig. 3A-B). One subject displayed focal radiotracer uptake in the culture positive discitis osteomyelitis [17]. Conversely, in the subject with culture negative Charcot arthropathy, the PET signal was similar between the affected foot and the contralateral joint.

### Biodistribution and Dosimetry

**Fig. 4:**
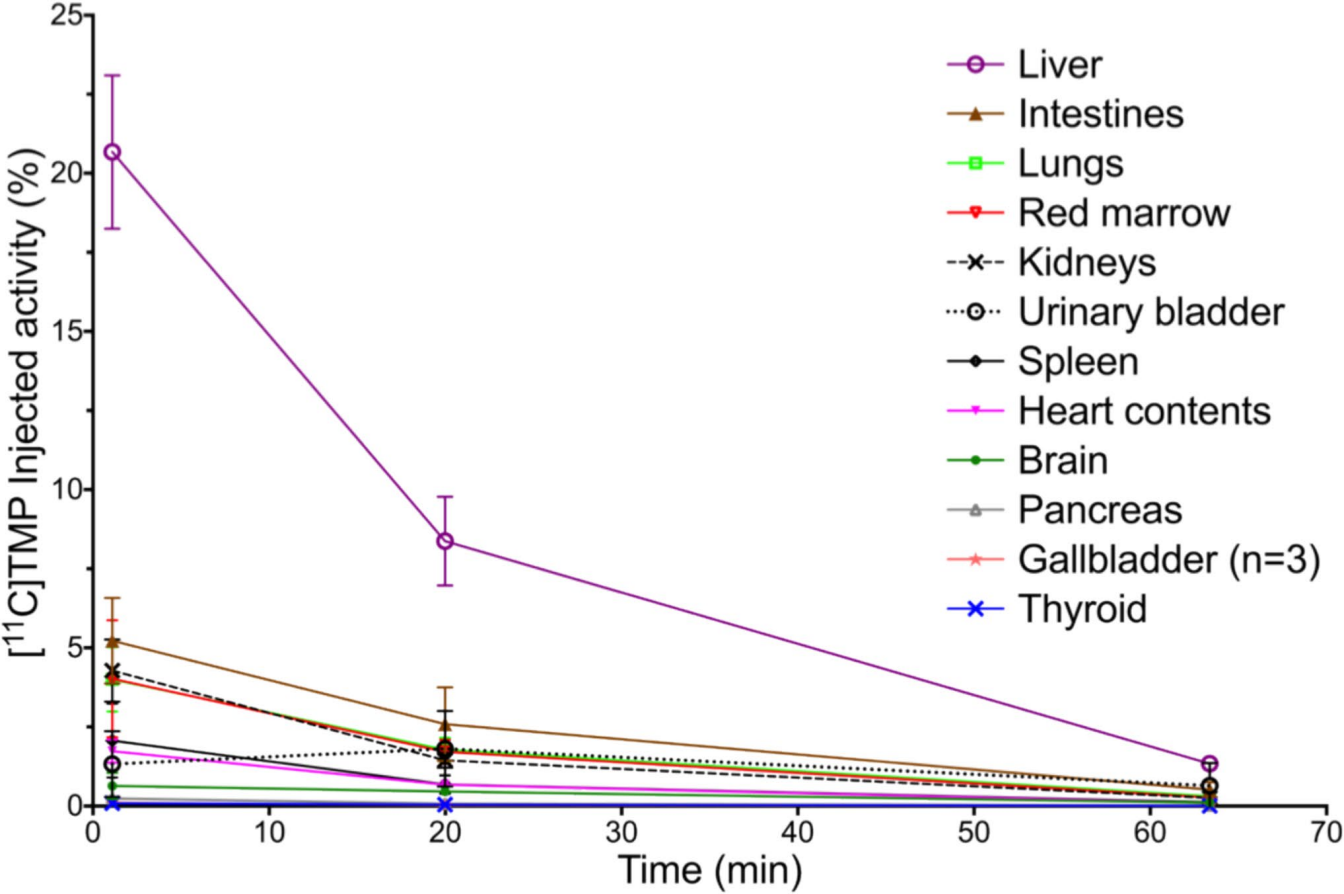
Mean [^11^C]TMP injected activity % in organs measured in the dosimetry cohort (± SD, *n* = 4). Subject 8 had a cholecystectomy and therefore no gallbladder uptake

Elevated uptake of [^11^C]TMP was noted in liver, kidneys, spleen, and bladder, consistent with the expected hepatic metabolism and urinary excretion of the tracer (Fig. 4) with estimated organ doses in Table 2 [24]. The highest mean organ absorbed doses were: liver (1.43E-02 mSv/MBq), kidneys (1.35E-02 mSv/MBq), spleen (1.18E-02 mSv/MBq), and urinary bladder wall (1.10E-02 mSv/MBq). The effective dose was 4.53E-03 mSv/MBq. The average dosimetry injection of 346 MBq (9.4 mCi) yielded an estimated effective dose of 1.6 mSv, and estimated absorbed doses of 4.9 mSv in the liver and 4.1 mSv in the blood-forming spleen, which are all below the Radioactive Drug Research Committee guidelines of a single 30 mSv dose and total annual dose of 50 mSv for blood-forming organs, allowing for the possibility of serial imaging. Physiologic uptake in the stomach and gastrointestinal tract was also observed, most visibly in the stomach at and after 90 min (Fig. 2**)**.

**Table 2:** Mean [^11^C]TMP absorbed dose estimates (*n* = 4)

Target organ	Mean ± SD (mSv/MBq)
Adrenals	3.47E-03 ± 1.13E-04
Brain	1.37E-03 ± 3.00E-04
Breasts	1.95E-03 ± 8.35E-05
Gallbladder wall*	5.00E-03 ± 4.43E-04
Lower large intestine wall	2.83E-03 ± 8.88E-05
Small intestine	9.29E-03 ± 2.33E-03
Stomach wall	2.83E-03 ± 6.34E-05
Upper large intestine wall	4.86E-03 ± 7.38E-04
Heart wall	4.66E-03 ± 3.36E-04
Kidneys	1.35E-02 ± 2.44E-03
Liver	1.43E-02 ± 1.58E-03
Lungs	5.62E-03 ± 1.05E-03
Muscle	2.25E-03 ± 6.29E-05
Ovaries	3.07E-03 ± 1.25E-04
Pancreas	5.27E-03 ± 2.20E-03
Bone (red marrow)	3.75E-03 ± 4.17E-04
Osteogenic cells	3.98E-03 ± 2.42E-04
Skin	1.73E-03 ± 6.60E-05
Spleen	1.18E-02 ± 4.64E-03
Testes	2.03E-03 ± 1.05E-04
Thymus	2.31E-03 ± 9.59E-05
Thyroid	5.96E-03 ± 1.73E-03
Urinary bladder wall	1.10E-02 ± 5.55E-03
Uterus	3.21E-03 ± 2.24E-04
Total Body	2.84E-03 ± 6.24E-05
Effective dose equivalent	6.08E-03 ± 3.49E-04
Effective dose	4.53E-03 ± 2.22E-04

*One subject had a cholecystectomy and was removed from gallbladder comparisons

## Discussion

In this cohort of 4 adult males, [^11^C]TMP displayed highest uptake in the liver, kidneys, spleen, and urinary bladder wall on PET imaging, consistent with metabolism of [^11^C]TMP in the liver and clearance through urinary excretion. Due to the background signal, evaluating infectious foci in these organs with [^11^C]TMP would be limited. Nonetheless, [^11^C]TMP rapidly clears from blood pool and shows low retention in many tissues (Figs. 2–4), including musculoskeletal tissues, potentially improving signal-to-background for lesion evaluation. These results also help to inform the existing literature on TMP’s applications as an antibiotic, including its common use in treating urinary tract infections. Additionally, while TMP radiotracers have demonstrated blood–brain permeability [25], [^11^C]TMP had low background uptake in the brain, indicating that radiolabeled TMP derivatives could be effective in characterizing and monitoring brain pathology. The absorbed doses in these organs allow repeat imaging, and scans suggest that image quality is adequate on clinical PET scanners at the 129-797 MBq radiotracer doses used in this study.

Only a small number of participants were recruited; however, we included subjects with infective and inflammatory processes to allow for a better characterization of the biodistribution of [^11^C]TMP in a cohort that represents the target population where this tracer could be used. Although we only had male patients in the biodistribution cohort, we have scanned female humans previously in a dynamic cohort, where we focused on infected lesions, usually in the setting of cystic fibrosis [8]. Additionally, due to the short-lived half-life of [^11^C], these dosimetry calculations are considered to be of lower priority than other longer-lived isotopes in tracer development [26]. Therefore, the key findings of this study are in the biodistribution in normal tissue and in the target patient population for early trials such as this study.

Imaging of infection with [^11^C]TMP has significant limitations, some due to the use of a short-lived radiotracer, and some inherent properties of bacterial infection imaging. The ~ 20-min half-life of ^11^C requires an on-site cyclotron for tracer production, as well as coordination between scanner availability and other priorities for patient care while under active treatment. Patients are also often on antibiotics, which may limit visualization of bacterial metabolic activity when imaging with [^11^C]TMP. Sites of infection may be relatively small or diffuse, presenting issues for partial volume effects and identification of primary locations to contrast between scans. Comparisons of imaging studies to sampled tissue cultures were limited by timing differences of days to months, during which infections and antibiotic regimens could, and did, change. This could be addressed by contemporaneous sampling and imaging, but would require either additional invasive sampling or research PET studies as an added consideration when coordinating patient care. While qualitative visualization of bacteria *in vivo* appears feasible, the somewhat limited contrast shown in the infections presented herein suggests that these limitations are not fully overcome by [^11^C]TMP, and that future development is needed.

Previously, we demonstrated that the vast majority of bacteria which cause clinical infection express DHFR and that therapeutic resistance to trimethoprim does not preclude the uptake and potential detection of these species with [^11^C]TMP [8]. More focused cohorts will be needed to examine the potential of [^11^C]TMP as an imaging agent for specific pathogens and clinical applications.

Additional applications for TMP are also in development, including the use of heterobifunctional TMP derivatives to regulate eDHFR tagged proteins (TMP PROTACs), which have potential for both basic research and therapeutics [27]. Companion PET imaging agents for these molecules can effectively act as a biomarker to monitor pharmacodynamic activity and may serve as an important tool for understanding their *in vivo* biodistribution and pharmacokinetics [28]. In addition, the use of eDHFR as a PET reporter gene in adoptive cell therapies is an area where many of the limitations of bacterial DHFR expression (low numbers of bacteria, partial volume effects, clearance of the bacteria by the immune system, etc.) may be overcome by the constitutive expression of eDHFR by the genetic vector delivering the therapeutic protein.

## Conclusion

[^11^C]TMP demonstrates low physiologic retention in many tissues, potentially enabling imaging in most tissues, including the musculoskeletal system. Our average dosimetry injected activity of 346 MBq (9.4 mCi) of [^11^C]TMP yielded an estimated average absorbed dose of 4.9 mSv in the highest uptake organ (liver) and an effective dose of 1.6 mSv, confirming that absorbed doses are low enough to allow serial [^11^C]TMP PET scans for monitoring of infection and assessment of response to antibiotic treatment. Additionally, as the image signal-to-background of [^11^C]TMP was limited given the *in vivo* protein target density of these patients’ infections, applications with higher potential target density such as eDHFR reporter gene-based cell tracking have substantial promise for future studies. Further development of TMP analogs or novel imaging markers of infection may also benefit from comparisons with [^11^C]TMP.

## Supplementary Information

Below is the link to the electronic supplementary material.

Supplementary file1 (DOCX 9497 KB)
