# Supplementary material for: First-in-Human Biodistribution and Dosimetry of [11C]Trimethoprim
Source: Mol Imaging Biol. 2025 Nov 22;28(1):60–7. doi: 10.1007/s11307-025-02064-7 (PMC12966202; doi:10.1007/s11307-025-02064-7)
Supplement: Supplementary file 1 — Supplementary file1 (DOCX 9497 KB) [file 11307_2025_2064_MOESM1_ESM.docx]

**SUPPLEMENTAL FIGURES**

**Supplemental Figure 1:** Maximum-intensity projections of [^11^C]TMP uptake for dosimetry subject 11 with high injected activity (797 MBq injection). Scan starts post-injection in upper left of panels. All images are decay corrected to time of injection (0 to 10 g/mL scale).

**
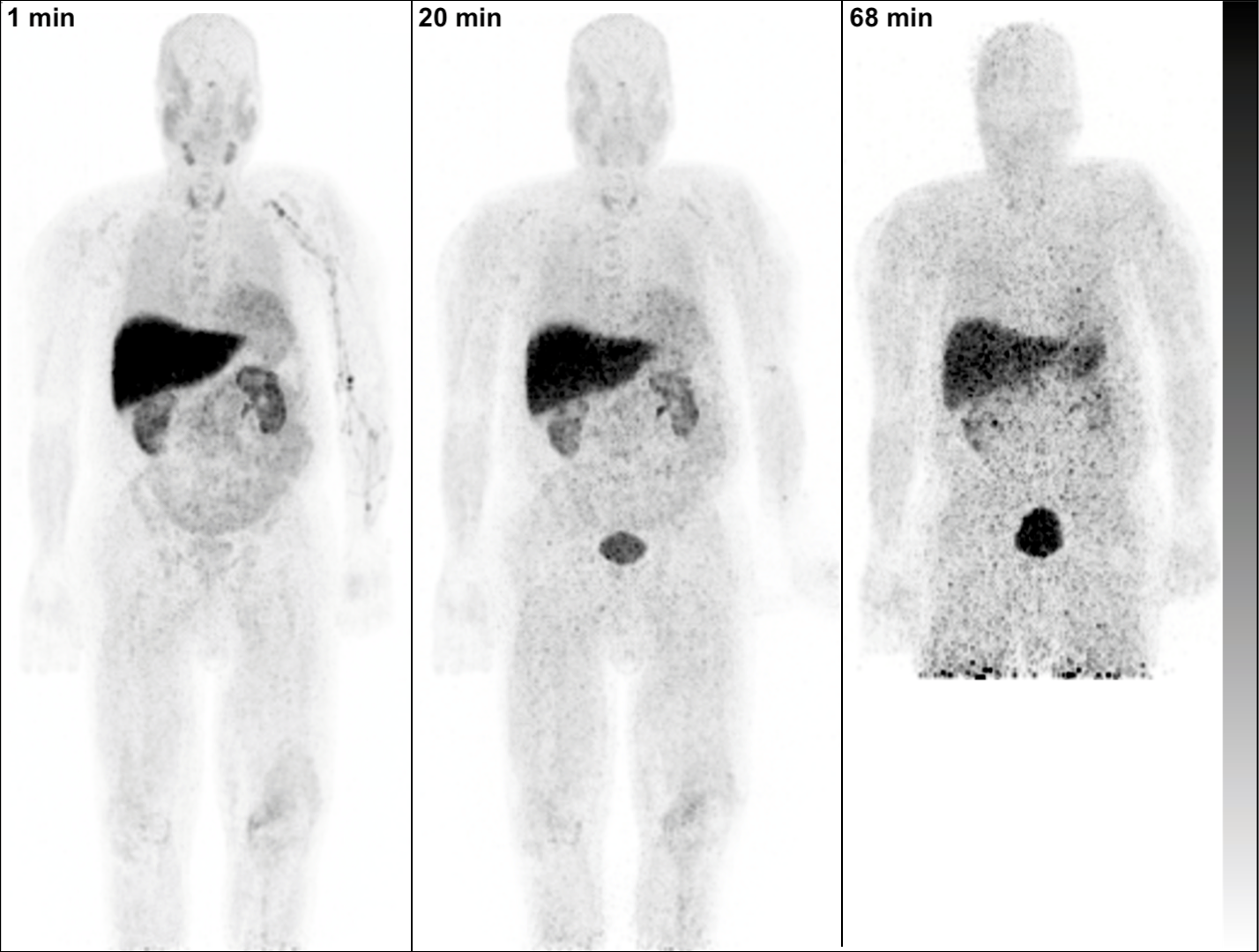
**

**Supplemental Figure 2:** Maximum intensity projections of [^11^C]TMP uptake for subject 8, with moderate injected activity (239 MBq injection). Scan starts post-injection in upper left of panels. All images are decay corrected to time of injection (0 to 10 g/mL scale).


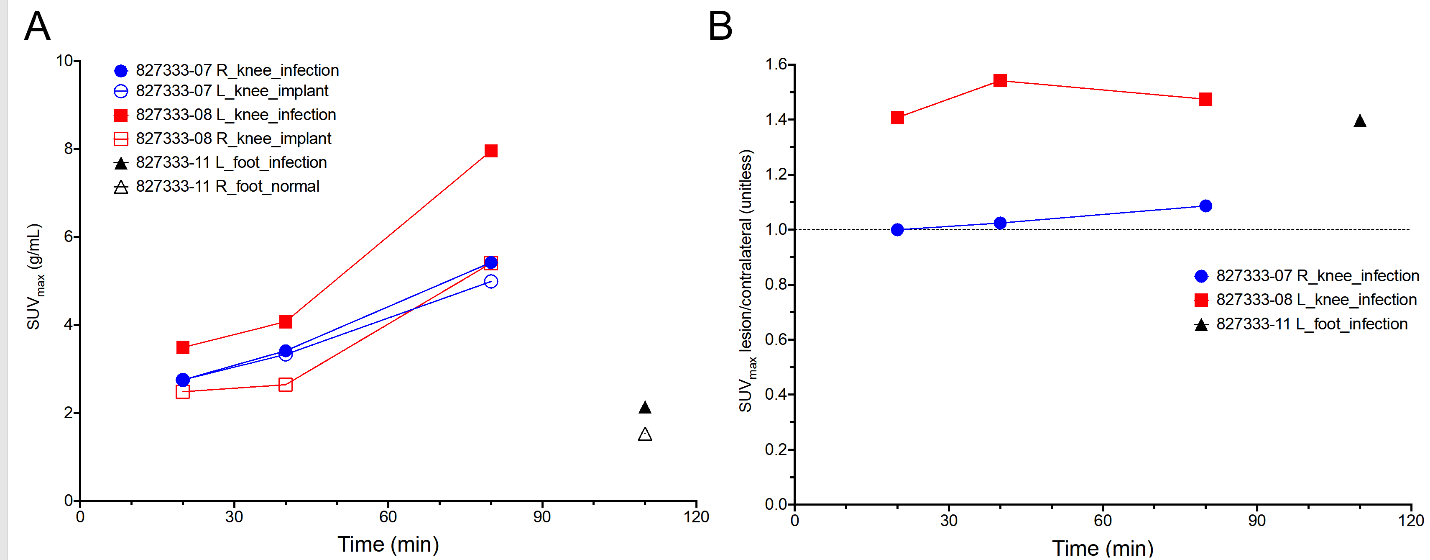


**Supplemental Figure 3**: **A**, [^11^C]TMP PET activity in suspected infections and contralateral tissues. **B**, lesion-to-contralateral tissue activity ratios.
